# Effectiveness of diterpene ginkgolides meglumine injection after endovascular therapy for cardioembolic stroke: a randomized controlled trial

**DOI:** 10.3389/fphar.2025.1707717

**Published:** 2026-01-08

**Authors:** Yiping Li, Kun Zhou, Jianan Wei, Xinmin Fu

**Affiliations:** 1 The Xuzhou Clinical College of Xuzhou Medical University, Xuzhou, China; 2 Department of Pulmonary and Critical Care Medicine, Northern Jiangsu People’s Hospital Affiliated to Yangzhou University, Yangzhou, China

**Keywords:** cardioembolic stroke, DGMI, diterpene ginkgolides meglumine injection, endovascular therapy, randomized controlled trial

## Abstract

**Background:**

Endovascular interventional therapy is an important treatment method for cardioembolic stroke. Diterpene Ginkgolides Meglumine Injection (DGMI) may serve as an adjunct to endovascular interventional therapy for acute ischemic stroke.

**Methods:**

Patients with cardioembolic stroke receiving endovascular treatment were recruited and randomly assigned in a 1:1 ratio to either the DGMI treatment group or the control group. After the procedure, all patients routinely received management of risk factors and standard medication. Patients in the treatment group additionally received DGMI 25 mg diluted in 250 mL normal saline, administered by intravenous drip once daily for 14 consecutive days. NIHSS scores, modified Rankin Scale (mRS) scores, and hematologic parameters were measured, with adverse reactions monitored throughout.

**Results:**

A total of 104 patients completed the study. After 14 days of treatment, HCT was significantly lower and PT was significantly higher in the treatment group ((*p* = 0.032 and *p* = 0.049, respectively). NIHSS and mRS scores in the treatment group were significantly lower than those in the control group at 14 days (*p* = 0.043 and *p* = 0.009, respectively). Similarly, the mRS score at 90 days was significantly lower in the treatment group (*p* = 0.009). The logistic regression analysis revealed that both group assignment and baseline NIHSS score were significantly associated with ΔNIHSS. A lower preoperative NIHSS score was associated with greater neurological improvement (*OR* = 0.893, 95% *CI* = 0.819–0.974, *p* = 0.011). Additionally, the DGMI group demonstrated superior effectiveness compared to the control (*OR* = 3.490, 95% *CI* = 1.077–11.316, *p* = 0.037).

**Conclusion:**

The combination of DGMI with endovascular therapy in patients with cardioembolic stroke is associated with reduced levels of coagulation biomarkers, improved neurological function, and better long-term prognosis, suggesting its potential as a complementary treatment following interventional procedures in clinical practice.

## 1 Introduction

Stroke ranks as the second most common cause of death globally, representing 11.6% of all mortality cases, with its incidence and mortality rates increasing annually (GBD, 2019 Stroke Collaborators, 2021), posing a severe threat to human life and health worldwide. Cardioembolic stroke accounts for over 20% of ischemic strokes (Yang H. et al., 2018). Compared to other ischemic stroke subtypes, it typically has a poorer prognosis and higher mortality rate, reaching up to 23% at 30 days, 22% at 1 year, and 50% within 1.5 years (Bhat et al., 2021), making it a serious global public health problem.

Treatment strategies for cardioembolic stroke primarily include conservative medical therapy, intravenous thrombolysis, and endovascular interventional therapy. Endovascular therapy, encompassing various procedures such as thrombectomy, balloon angioplasty, and stenting, has been proven superior to medical therapy alone (Jovin et al., 2022). It can rapidly recanalize occluded vessels, restore cerebral blood flow perfusion, and salvage the ischemic penumbra. However, postoperative brain tissue still suffers further neuronal damage due to ischemia-induced oxidative stress and inflammatory responses (Zhao Y. et al., 2022), which are difficult to address with conventional treatment regimens. Therefore, exploring new neuroprotective agents to improve surgical outcomes is necessary.

Ginkgo biloba leaf is a traditional Chinese medicine that improves blood circulation, and its extracts are widely used globally for treating various cardiovascular and cerebrovascular diseases (Xiao et al., 2022). Diterpene Ginkgolides Meglumine Injection (DGMI) is derived through standardized extraction of Ginkgo biloba leaf and belongs to a novel class of Chinese medicine preparations. Its active components mainly include ginkgolide A (GA), ginkgolide B (GB), and ginkgolide K (GK) (Fan et al., 2021). Studies have shown that DGMI can inhibit platelet aggregation, exert anti-inflammatory and antioxidant effects, and reduce cerebral edema. Its clinical use in combination with Western medicine can better improve treatment efficacy (Yan et al., 2023). However, most studies have focused on patients with mild to moderate cerebral infarction, and clinical data on its combination with endovascular therapy are scarce. Therefore, this study aims to evaluate the efficacy of DGMI in patients with cardioembolic stroke after endovascular therapy.

## 2 Materials and methods

### 2.1 Study design and participants

This trial was an open-label, randomized controlled study with blinded endpoint assessment. Patients with cardioembolic stroke receiving endovascular treatment at Xuzhou Central Hospital between January 2023 and May 2025 were recruited and randomly assigned in a 1:1 ratio to either the DGMI treatment group or the control group. According to ethical requirements, patients sign informed consent forms, and endpoint blinding is adopted. The outcome indicators, namely NIHSS and mRS Scores, are blinded and evaluated by neurologists who are unaware of the grouping situation and treatment measures. The study was approved by the hospital’s ethics committee (Ethics Approval No.: XZXY-LK-20220707–059) and registered at ClinicalTrials.gov (registration number NCT02526225).

All patients were placed in the supine position preoperatively, and the anesthesia method was selected based on consciousness level. Patients with good consciousness who could cooperate received local anesthesia; those with impaired consciousness who could not cooperate received general anesthesia. Most patients underwent endovascular therapy via the modified Seldinger technique under local anesthesia, involving puncture of one femoral artery and placement of a 6F or 8F arterial sheath. Full cerebral angiography was performed first. If vascular occlusion or subtotal occlusion suitable for intervention was found, endovascular therapy was initiated. The culprit vessel was located based on angiographic findings, and an appropriate thrombectomy device (such as Solitaire FR or the domestic Sinomed Intracranial Thrombus Removal Device) was selected, deployed within the target vessel, and thrombectomy performed. Post-procedural angiography was conducted for mTICI scoring. If mTICI grade reached 2b or 3, indicating successful recanalization, the femoral artery puncture site was closed, and the procedure ended. If mTICI grade was below 2b, further intracranial balloon angioplasty or cerebral stenting (using devices such as Solitaire AB, Solitaire FR detachable stents, or dedicated intracranial stents like Apollo) was performed to restore vessel morphology and achieve mTICI grade 2b or 3. If vascular recanalization cannot be achieved even after rescue measures, individualized neuro-surgical treatment or conservative management will be adopted based on the patient’s specific condition. Patients were screened and randomized after confirming that the mTICI grade reached 2b or 3.

Randomization Sequence Generation: This study utilized a block randomization method. An independent statistician, who was not involved in the trial, generated the randomization sequence using SAS software, allocating participants in a 1:1 ratio to either the treatment group or the control group. Allocation Concealment: The generated randomization sequence was implemented via a central randomization system. Once a patient met all eligibility criteria, the system automatically assigned group allocation according to the predetermined sequence. Furthermore, the randomization list remained concealed from investigators, patients, and outcome assessors throughout the entire enrollment period.

All patients received routine management of high-risk factors and standardized guideline-directed medical therapy postoperatively: anticoagulation therapy was initiated within 2 weeks after thrombectomy for patients with cardioembolic stroke; statin therapy was initiated immediately and maintained long-term in all patients; the target systolic blood pressure was maintained below 180 mmHg postoperatively, with a long-term goal of systolic blood pressure below 140 mmHg thereafter; dehydration therapy was administered only to patients with clear clinical or radiological evidence of cerebral edema. Patients in the treatment group additionally received DGMI 25 mg diluted in 250 mL normal saline, administered by intravenous drip once daily for 14 consecutive days. To ensure the single-variable principle of the study and accurately evaluate the efficacy of DGMI.

### 2.2 Inclusion and exclusion criteria

Inclusion criteria: (1) Age ≥18 years; (2) Acute ischemic stroke, with acute large vessel occlusion confirmed by CTA or MRA, and consistent with cardioembolic etiology of the culprit vessel (Garkina et al., 2021); (3) Presence of high-risk cardiac conditions on cardiac examination, such as atrial fibrillation, endocarditis, patent foramen ovale, etc.; (4) Preoperative National Institutes of Health Stroke Scale (NIHSS) score ≥6; (5) No significant disability before onset (pre-stroke mRS 0–1); (6) Puncture treatment expected to commence within 24 h of onset, with randomization completed simultaneously; (7) Preoperative Alberta Stroke Program Early CT Score (ASPECTS) ≥6; (8) Presence of salvageable ischemic brain tissue measured by CTP imaging using RAPID automated software; (9) Postoperative modified Thrombolysis in Cerebral Infarction (mTICI) grade ≥2b; (10) Written informed consent was provided by all participants or their legally designated proxies.

Exclusion criteria: (1) Angiographic evidence of large artery atherosclerosis inconsistent with cardioembolism; (2) Received intravenous thrombolysis before the procedure; (3) Intracranial hemorrhage or subarachnoid hemorrhage shown on CT or MRI; (4) Absence of salvageable ischemic tissue according to automated CTP software measurement; (5) Known severe allergy to iodine contrast media; (6) Tortuous access or other difficulties preventing the device from reaching the target vessel; (7) Active bleeding or known history of bleeding tendency; (8) Gastrointestinal or urinary tract bleeding within the last 3 weeks; (9) Blood glucose <2.7 mmol/L or >22.2 mmol/L; (10) Blood pressure exceeding 185/110 mmHg uncontrolled by medication; (11) Known dementia or psychiatric illness preventing neurological assessment; (12) Life expectancy less than 1 year; (13) Severe cardiac, hepatic, or renal insufficiency or other severe advanced diseases deemed unsuitable for the study by the investigator; (14) Participation in other drug or device clinical trials.

### 2.3 Observation indicators and clinical efficacy evaluation

NIHSS assessments were conducted at baseline and on day 14 post-treatment, while modified Rankin Scale (mRS) evaluations were performed at pretreatment, 14-day, and 3-month follow-up intervals. (2) Levels of hematocrit (HCT), prothrombin time (PT), thrombin time (TT), and activated partial thromboplastin time (APTT) before and 14 days after treatment. (3) Monitoring adverse reactions within 14 days after medication administration, including any intracerebral hemorrhage, symptomatic intracerebral hemorrhage defined by the National Institute of Neurological Disorders and Stroke (NINDS) criteria (National Institute of Neurological Disorders and Stroke rt-PA Stroke Study Group, 1995), extracranial bleeding events, serious adverse events, and 90-day mortality.

### 2.4 Primary and secondary endpoints

The primary outcome measure was the degree of neurological enhancement, as evaluated through NIHSS score comparisons between baseline and day 14 post-intervention. Effectiveness was defined as a reduction in NIHSS score (ΔNIHSS) >4 points (NIHSS_day0_-NIHSS_day14_ >4) or an NIHSS _day14_ score below four points. In 1995, the National Institute of Neurologic Disorders and Stroke (NINDS) study on tissue plasminogen activator in acute ischemic stroke evaluated neurological outcomes by dichotomizing the ΔNIHSS based on a reduction of >4 points, which was defined as a reliable indicator of post-treatment neurological improvement (Hoffman and Schriger, 2009). In 2020, this same criterion was adopted in a clinical trial data analysis investigating the efficacy of intravenous ginkgolide in Chinese patients with ischemic stroke (Dong et al., 2020).

The secondary endpoints included changes in hematologic parameters, specifically alterations in HCT, PT, TT, and APTT after 14 days of treatment.

### 2.5 Sample size calculation

The sample size was calculated using PASS15 statistical software, employing the sample size estimation method for a randomized controlled trial. In this study, the sample size was determined based on the primary endpoint of neurological improvement rate. Based on our preliminary clinical experience, we hypothesized that the neurological improvement rate would be 67% in the DGMI group after treatment, compared to 40% in the control group, with α = 5% and β = 20%. The calculation indicated that a sample size of 100 participants was required. Assuming a 20% dropout rate, a total of 120 participants (60 participants per group) were needed for this study.

### 2.6 Statistical analysis

Statistical analyses were conducted using SPSS 27.0 software. Count data are expressed as *n* (%), using the chi-squared test for comparisons. The normality of continuous variables was assessed using the Shapiro-Wilk test. Data with a normal distribution are expressed as the mean ± standard deviation (SD), analyzed using the *t*-test. Data with a non-normal distribution are expressed as median and interquartile range (IQR); comparisons between groups used the Mann-Whitney *U* test, and intragroup comparisons used the Wilcoxon signed-rank test. ΔNIHSS was taken as the dependent variable. Univariate and multivariate binary logistic regression analyses were used to explore factors influencing effectiveness. A *p* value < 0.05 was considered statistically significant.

## 3 Results

### 3.1 Baseline characteristics

**FIGURE 1:**
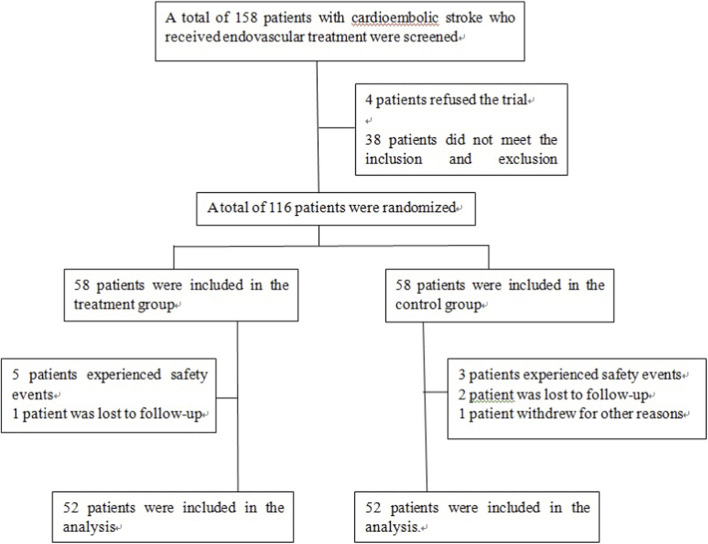
Flow diagram of the study.

From January 2023 to May 2025, 104 patients were enrolled, with 52 patients each in the treatment and control groups. A flow diagram of the study is shown in Figure 1. There were no statistically significant differences between the two groups in terms of age, gender, past medical history, smoking history, drinking history, lesion location, onset-to-puncture time, preoperative ASPECTS score, number of thrombectomy procedures, mTICI grade, or the use of rescue measures like balloon angioplasty or stenting (*p* > 0.05). The baseline characteristics are shown in Table 1.

**TABLE 1:** Baseline characteristics of all patients.

Characteristics	Treatment group (*n* = 52)	Control group (*n* = 52)	*t*/*Z*/*χ* ^*2*^	*P*
Age, mean (SD), y	68.83 ± 9.84	68.98 ± 11.53	−0.073	0.942
Male, *n* (%)	27 (51.92)	32 (61.54)	0.979	0.322
Smoking, *n* (%)	26 (50.00)	29 (55.77)	0.347	0.556
Drinking, *n* (%)	24 (46.15)	29 (55.77)	0.962	0.327
Hypertension, *n* (%)	36 (69.23)	37 (71.15)	0.046	0.830
Diabetes, *n* (%)	14 (26.92)	12 (23.08)	0.205	0.651
Hyperlipidemia, *n* (%)	10 (19.23)	12 (23.08)	0.231	0.631
Stroke history, *n* (%)	22 (42.31)	18 (34.62)	0.650	0.420
Lesion location, *n* (%)			0.780	0.780
Anterior circulation	44 (84.62)	45 (86.54)		
Posterior circulation	8 (15.38)	7 (13.46)		
Onset to puncture, median (IQR), hr	5.00 (3.50–6.50)	5.25 (3.50–8.38)	0.404	0.686
ASPECTS, median (IQR)	7 (6–7)	7 (6–8)	1.859	0.063
Number of thrombectomy, *n* (%)			0.053	0.819
1	40 (76.92)	39 (75.00)		
>1	12 (23.08)	13 (25.00)		
mTICI, *n* (%)			0.295	0.587
2b	7 (13.46)	9 (17.31)		
3	45 (86.54)	43 (82.69)		
Intracranial angioplasty/stenting, *n* (%)	22 (42.31)	23 (44.23)	0.039	0.843

Abbreviations: ASPECTS, Alberta stroke program early CT, score; mTICI, modified thrombolysis in cerebral infarction; SD, standard deviation; IQR, interquartile range.

### 3.2 Hematologic parameters

Before treatment, there were no significant differences in HCT, PT, TT, and APTT levels between the two groups (*p* > 0.05). After 14 days of treatment, HCT decreased in both groups compared to pre-treatment levels (*p* < 0.001), and was significantly lower in the treatment group (*p* = 0.032); PT increased in both groups (*p* < 0.001), and was significantly higher in the treatment group (*p* = 0.049). Compared to pre-treatment levels, after 14 days, TT and APTT increased in the treatment group (*p* = 0.013 and *p* < 0.001, respectively), while in the control group, TT did not change significantly (*p* = 0.942) and APTT increased (*p* < 0.001). The differences between the two groups after treatment for TT and APTT were not statistically significant (*p* = 0.058 and *p* = 0.658, respectively) (Table 2).

**TABLE 2:** Hematologic parameters of all patients before and after 14 days of treatment.

Characteristics	Treatment group (*n* = 52)	Control group (*n* = 52)	*t*	*P*
HCT, mean (SD), %				
Before	40.79 ± 4.32	41.43 ± 3.72	−0.798	0.427
After	36.97 ± 4.84	38.80 ± 3.72	−2.170	**0.032**
*t* (Intra)	9.599	11.756	​	​
*P* (Intra)	**<0.001**	**<0.001**	​	​
PT, mean (SD), s				
Before	11.97 ± 0.72	11.91 ± 0.83	0.365	0.716
After	12.90 ± 1.18	12.50 ± 0.86	1.994	**0.049**
*t* (Intra)	−6.549	−7.786	​	​
*P* (Intra)	**<0.001**	**<0.001**	​	​
TT, mean (SD), s				
Before	15.99 ± 1.16	16.09 ± 0.99	−0.473	0.637
After	16.70 ± 1.79	16.08 ± 1.51	1.919	0.058
*t* (Intra)	−2.577	0.073	​	​
*P* (Intra)	**0.013**	0.942	​	​
APTT, mean (SD), s				
Before	25.15 ± 2.54	25.94 ± 2.12	−1.718	0.089
After	28.69 ± 3.37	28.43 ± 2.49	0.444	0.658
*t* (Intra)	−7.259	−10.508	​	​
*P* (Intra)	**<0.001**	**<0.001**	​	​

Abbreviations: HCT, hematocrit; PT, prothrombin time; TT, thrombin time; APTT, activated partial thromboplastin time; SD, standard deviation; Intra, intragroup comparison before vs. after. The bolded values indicate that p < 0.05.

### 3.3 Clinical outcomes

**FIGURE 2:**
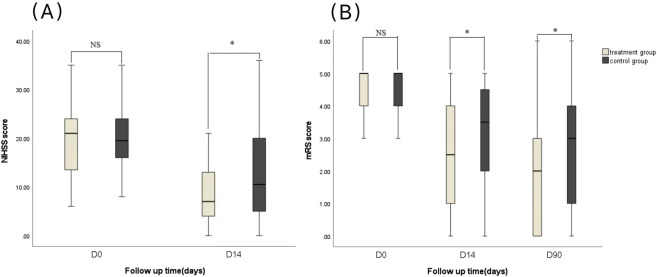
Clinical outcomes of all patients. **(A)** National institute of health stroke scale score (NIHSS). **(B)** Modified rankin score (mRS). ^NS^ *p* ≥ 0.05, ^*^ *p* < 0.05, compared with the control group.

Regarding clinical outcomes, there were no significant differences in NIHSS and mRS scores between the two groups before treatment (*p* > 0.05). After 14 days of treatment, both NIHSS and mRS scores decreased in both groups (*p* < 0.001). The scores in the treatment group were significantly lower than those in the control group at 14 days (*p* = 0.043 and *p* = 0.009, respectively). Similarly, the mRS score at 90 days was significantly lower in the treatment group (*p* = 0.009) (Table 3; Figure 2).

**TABLE 3:** Clinical outcomes of all patients.

Characteristics	Treatment group (*n* = 52)	Control group (*n* = 52)	*Z*	*P*
NIHSS, median (IQR)				
Before	21.00 (13.25–24.00)	19.50 (16.00–24.00)	0.706	0.480
Day 14	7.00 (4.00–13.00)	10.50 (5.00–20.00)	2.022	**0.043**
*Z* (Intra)	−5.448	−4.836	​	​
*P* (Intra)	**<0.001**	**<0.001**	​	​
mRS, median (IQR)				
Before	5.00 (4.00–5.00)	5.00 (4.00–5.00)	1.619	0.105
Day 14	2.50 (1.00–4.00)	3.50 (2.00–4.75)	2.623	**0.009**
*Z* (Intra)	−5.620	−5.175	​	​
*P* (Intra)	**<0.001**	**<0.001**	​	​
mRS at day 90, median (IQR)	2.00 (0.00–3.00)	3.00 (1.00–4.00)	2.602	**0.009**

Abbreviations: NIHSS, national institutes of health stroke scale; mRS, modified rankin scale; IQR, interquartile range; Intra, Intragroup comparison before vs. after (day 14 for NIHSS/mRS). The bolded values indicate that p < 0.05.

To investigate factors influencing neurological improvement, univariate binary logistic regression analysis was performed first. The results showed that ΔNIHSS was significantly associated with preoperative NIHSS score (*OR* = 0.941, 95% *CI* = 0.885–0.999, *p* = 0.048) and group assignment (*OR* = 2.671, 95% *CI* = 1.033–6.910, *p* = 0.043), but not with other factors. All clinically relevant covariates were included in the multivariate binary logistic regression analysis. Results showed that both group assignment and baseline NIHSS score were significantly associated with ΔNIHSS. A lower preoperative NIHSS score was associated with greater neurological improvement (*OR* = 0.893, 95% *CI* = 0.819–0.974, *p* = 0.011). Additionally, the DGMI group demonstrated superior effectiveness compared to the control (*OR* = 3.490, 95% *CI* = 1.077–11.316, *p* = 0.037) (Table 4).

**TABLE 4:** Logistic regression analysis of the factors associated with clinical improvement (ΔNIHSS >4) after 14 days of therapy.

Variables	Univariate analysis		Multivariate analysis	
	OR (95% CI)	*P*	OR (95% CI)	*P*
Age, y	0.971 (0.929, 1.015)	0.188	0.992 (0.941, 1.046)	0.758
Male	1.287 (0.522, 3.175)	0.584	1.848 (0.436, 7.827)	0.404
Time from onset to puncture, hr	0.923 (0.815, 1.045)	0.207	0.901 (0.772, 1.052)	0.186
NIHSS score at baseline	0.941 (0.885, 0.999)	**0.048**	0.893 (0.819, 0.974)	**0.011**
mRS score at baseline	1.002 (0.570, 1.761)	0.996	1.838 (0.785, 4.300)	0.161
ASPECTS	0.714 (0.436, 1.171)	0.182	0.714 (0.390, 1.307)	0.275
Number of thrombectomy procedures	0.726 (0.404, 1.303)	0.283	0.611 (0.281, 1.329)	0.214
mTICI	0.580 (0.178, 1.893)	0.366	0.787 (0.185, 3.341)	0.746
Grouping (treatment vs. control)	2.671 (1.033, 6.910)	**0.043**	3.490 (1.077, 11.316)	**0.037**
Hypertension	0.873 (0.331, 2.303)	0.783	1.160 (0.244, 5.520)	0.852
Diabetes	0.494 (0.152, 1.601)	0.240	1.023 (0.218, 4.807)	0.977
Hyperlipidemia	0.431 (0.116, 1.599)	0.208	0.318 (0.065, 1.553)	0.157
Stroke history	0.542 (0.203, 1.446)	0.221	0.614 (0.172, 2.198)	0.454
Lesion location	1.725 (0.528, 5.632)	0.366	2.284 (0.528, 9.885)	0.269
Intracranial angioplasty/stenting	1.040 (0.420, 2.575)	0.933	1.496 (0.483, 4.633)	0.485

Abbreviations: NIHSS, national institutes of health stroke scale; mRS, modified rankin score; OR, odds ratio; CI, confidence interval. The bolded values indicate that p < 0.05.

### 3.4 Safety analysis

During hospitalization within 14 days after medication administration, any intracerebral hemorrhage was assessed via CT, with 2 cases identified in the treatment group and 3 cases in the control group. Symptomatic intracerebral hemorrhage, evaluated according to the National Institute of Neurological Disorders and Stroke (NINDS) criteria, was detected in 1 case in the treatment group and 0 cases in the control group. No extracranial bleeding events or serious adverse events were observed. Only two mild adverse reactions were reported: one patient experienced gastrointestinal symptoms, including nausea and abdominal bloating, while another presented with facial flushing, the symptoms resolved after discontinuation of the medication. The patients who experienced adverse reactions discontinued the trial and were excluded from the analysis. At the 90-day follow-up, mortality was recorded based on patient revisit examinations and telephone follow-ups, with rates of 7.69% in the treatment group and 11.54% in the control group. The difference was not statistically significant (*χ* ^*2*^ = 0.443, *p* = 0.506).

## 4 Discussion

Cardioembolic stroke, as an important subtype of ischemic stroke, is characterized by high disability rate and high mortality. Its incidence is increasing with global population aging (Prust et al., 2024). Endovascular therapy techniques, including mechanical thrombectomy, balloon angioplasty, and stenting, directly remove thrombi and rapidly recanalize occluded large vessels. However, modern medicine continues to explore ways to further enhance postoperative treatment effectiveness and improve prognosis. Traditional Chinese medicine, known for its multi-target and sustained effects, has shown unique advantages in combined therapy with Western medicine in recent years.

This study found that patients receiving DGMI combined with endovascular therapy had lower NIHSS and mRS scores and significantly higher clinical effectiveness at 14 days, indicating that DGMI can effectively improve neurological function and long-term prognosis. This result has been confirmed in previous studies. A meta-analysis of Ginkgo biloba-related drugs found that they significantly improved clinical effectiveness rates, functional independence, and neurological deficits in stroke patients (Li et al., 2023). Studies on DGMI also showed that, as a multi-target neuroprotective agent, it exerts neuroprotective and reparative effects through multiple pathways, increasing the proportion of favorable clinical outcomes (Zhang et al., 2023). Therefore, this study suggests that DGMI can serve as a supplementary therapy after endovascular treatment for ischemic stroke.

The main components of DGMI are GA, GB, and GK, all extracted from Ginkgo biloba leaves. Modern pharmacology has identified their multiple mechanisms of action, synergistically contributing to neuroprotection. GA improves tissue ischemia and inhibits neuronal apoptosis by repairing cholinergic nerves and blocking pro-apoptotic factor activation (Li et al., 2024). GB-related animal experiments found that it enhances the antioxidant defense system, reduces damage to antioxidant genomes, counteracts oxidative stress (Feng et al., 2019), inhibits inflammatory mediators, modulates the equilibrium of pro- and anti-inflammatory responses within the cerebral environment, and protects blood-brain barrier permeability (Zhang X. et al., 2021). It also inhibits apoptosis and achieves neuroprotection by activating PKA, Akt, ERK1/2, and the G protein-coupled receptor EP4 (Yang et al., 2021). GK possesses the strongest antiplatelet aggregation function and can reduce cerebral edema, tissue necrosis, and reperfusion injury after cerebral infarction (Dong et al., 2021). A fundamental study using Bunium persicum as a research model, which delves into the key genes and biosynthetic pathways involved in terpenoid production, contributes to understanding the clinical efficacy of DGMI from a biological perspective (Samandari-Bahraseman et al., 2025).

Previous studies have confirmed DGMI’s antiplatelet aggregation function, which most scholars believe is mediated through specific antagonism of the platelet-activating factor receptor (PAFR) (Xu et al., 2025). The pathway associated with PAF plays a pivotal role in mediating neuroinflammatory processes throughout the initiation and progression of ischemic stroke, and its inhibition by DGMI helps reduce the infarct volume in acute ischemic stroke. Other studies indicate that DGMI may affect arachidonic acid metabolism, inhibiting the classic arachidonic acid pathway of platelet activation, thereby enhancing the antiplatelet aggregation effect of drugs that act by inhibiting arachidonic acid (Chen et al., 2023).

However, the coagulation system in cardioembolic ischemic stroke also undergoes complex functional changes. The disruption of the balance between coagulation and anticoagulation often exacerbates thrombus formation and ischemic brain injury. Regulating coagulation function remains a key target for improving treatment efficacy in cardioembolic stroke and a core starting point for the development of anticoagulants and optimization of treatment regimens (Ye et al., 2021). Therefore, this study evaluated the impact of DGMI on coagulation function in patients with cardioembolic ischemic stroke using coagulation parameters. The results showed increases in PT, TT, and APTT after DGMI treatment, with the increase in PT being statistically significant compared to the control group. PT is an important clinical indicator for monitoring the extrinsic coagulation pathway, reflecting the functional efficiency of this system. In this study, the prolongation of PT indicates that DGMI exerts a certain anticoagulant effect. However, the absence of a significant difference in the number of recorded safety events between the two groups suggests that the anticoagulant activity of DGMI is limited and relatively mild. In clinical practice, PT can serve as a rough indicator for assessing bleeding risk in patients taking rivaroxaban (Zhang et al., 2016), and mild prolongation of PT is not associated with an increased incidence of bleeding events (Yuan et al., 2023). A recent retrospective study found that the risk of major adverse cardiac and cerebrovascular events (MACCE) is associated with the estimated glucose disposal rate (eGDR). Specifically, in populations with prediabetes and non-diabetes, an increase in eGDR was associated with a decreased incidence of MACCE. Clinically, eGDR may serve as a useful indicator for identifying individuals at high risk of cardiovascular and cerebrovascular events, enabling early intervention (Shojaei et al., 2025). Another study on the effect of DGMI on coagulation function indicated that fibrinogen (FIB) and D-dimer (DD) levels also decreased after treatment (Zhang et al., 2024), suggesting that DGMI can regulate hypercoagulability, improve cerebral blood flow efficiency, thereby promoting normal microcirculation in brain tissue and maintaining its physiological function. Moreover, DGMI’s effect on blood status is relatively mild and does not significantly increase bleeding risk, making it a relatively safe treatment option in clinical practice (Huang et al., 2025). Overall, DGMI can improve blood flow by exerting both antiplatelet aggregation and anticoagulant functions.

Furthermore, this study found that HCT after DGMI treatment was significantly lower than in the control group, suggesting DGMI can effectively reduce patients’ HCT levels. HCT is a hemorheological indicator representing the volume percentage of red blood cells in blood. Elevated HCT increases blood flow resistance, interferes with hemodynamic stability, affects blood circulation and cerebral perfusion, leading to cerebral ischemia and hypoxia, damaging the nervous system, and worsening cerebral infarction (Roh et al., 2024). HCT may also activate adenosine diphosphate, inducing platelet spreading and adhesion, increasing the risk of thrombosis and recurrent cerebral infarction (Karino and Goldsmith, 1984). Recent studies found that HCT is closely related to the prognosis of cerebral infarction; elevated HCT increases the risk of death and poor outcomes, and HCT levels can predict unfavorable outcomes in patients with acute ischemic stroke (Cui et al., 2024). This study used HCT as an observation indicator and found that DGMI can reduce HCT levels in patients with cardioembolic ischemic stroke. HCT may become an important marker for evaluating the efficacy and prognosis of cerebral infarction in the future. A large prospective study indicated that elevated HCT is associated with an increased risk of atherosclerotic cardiovascular disease events in the general population. Among patients with familial hypercholesterolemia, HCT shows a positive correlation with the incidence of major adverse cardiovascular events (MACE), particularly when HCT levels exceed the median, where the trend toward increased MACE becomes more pronounced, and event-free survival probability is significantly lower (Paquette et al., 2021). Additionally, a study focusing on the Chinese population demonstrated that higher HCT levels are linked to an elevated risk of stroke and show no association with intracranial hemorrhage (Yang R. et al., 2018). Similarly, in other systemic conditions such as retinal artery occlusion, elevated HCT also contributes to an increased risk (Lai et al., 2022).

To our knowledge, there are currently very few studies on the efficacy of DGMI in patients undergoing endovascular therapy. Endovascular treatment often causes vascular wall injury, which more readily activates platelet aggregation and triggers a series of more complex hematological responses (Mereuta et al., 2022). Therefore, different from patients receiving only conventional medical therapy, the medication regimen after endovascular therapy deserves separate discussion. This study observed that group assignment was an factor influencing neurological function, with the group receiving postoperative DGMI treatment having higher effectiveness than the control group. DGMI’s multifaceted effects on acute ischemic stroke may explain it. Previous studies indicate that DGMI can reduce inflammatory markers in the blood (Mousavi et al., 2022). Post-stroke energy metabolism disorder in brain tissue is a major cause of brain cell damage, closely related to inflammatory responses and oxidative stress (Zhang Q. et al., 2021). The reduction of inflammatory factors inhibits the series of damages caused by oxygen free radicals, producing a cerebral protective effect. Another cell experiment confirmed that active components in DGMI can affect myelin stability, inhibit demyelination, promote remyelination, improve apoptosis after cerebral infarction, and reduce brain water volume and infarct area in experimental animals, thereby inhibiting further postoperative ischemic brain injury, protecting nerves, and promoting neurological recovery (Wang et al., 2022). From a practical perspective, DGMI can improve the quality of life, enhance mobility, alleviate disease suffering, and reduce social care burden for stroke patients (Tian et al., 2024a). Some scholars even point out that cognitive function also showed significant improvement in acute stroke patients treated with DGMI, especially in language, visual, and executive functions, with similar improvements observed in Alzheimer’s disease patients (Tian et al., 2024b), suggesting DGMI may potentially inhibit vascular cognitive impairment caused by cerebral ischemia. Therefore, combined DGMI treatment after endovascular intervention for cerebral infarction has beneficial effects on patients’ neurological function, quality of life, and cognitive level.

Clinical treatment for cardioembolic ischemic stroke is never monotherapy. With in-depth research on Ginkgo preparations, the combined effects of DGMI with other drugs are being gradually revealed. Studies indicate that thrombolytic drug use can lead to brain tissue metabolic disorders and blood-brain barrier disruption (Zhao H. et al., 2022), while combination with DGMI compensates for this side effect and improves the efficacy of thrombolytics (Dong et al., 2020). Combination therapy with aspirin can also reduce adverse reactions to antiplatelet drugs, lower drug resistance (Zhao et al., 2021), reduce recurrence probability, without increasing bleeding events (Han et al., 2023). It also demonstrates cost-effectiveness in the long term, alleviating patients’ economic burden (Xiang et al., 2021). Simultaneous use with other Chinese medicine preparations, such as Butylphthalide, also shows synergistic effects (Xin-Shuai et al., 2024). It demonstrates good efficacy in acute ischemic stroke across different age groups (Zhang et al., 2022).

This study has certain limitations: This study is a single-center trial with a relatively homogeneous enrolled population, which may not fully represent the spectrum of all relevant clinical cases, thereby limiting the generalizability of the conclusions. Second, the modest sample size reduces the statistical power of the study and the ability to detect differences in safety outcomes. Finally, this was an open-label trial; although blinded endpoint assessment was implemented, the non-blinded nature of the treatment process may still introduce potential bias. Further validation through multicenter, large-sample, randomized controlled trials is still required to confirm these findings and provide more robust evidence for consolidation therapy after endovascular treatment in clinical practice.

## 5 Conclusion

In summary, the combination of DGMI with endovascular therapy in patients with cardioembolic stroke is associated with reduced levels of coagulation biomarkers, improved neurological function, and better long-term prognosis, suggesting its potential as a complementary treatment following interventional procedures in clinical practice.

## Data Availability

The data presented in the study are deposited in the Figshare repository, accession number 10.6084/m9.figshare.30911030 (https://doi.org/10.6084/m9.figshare.30911030).
